# Confirming the Reliability and Validity of the Sexual Minority Adolescent Stress Inventory in a National Sample of Sexual Minority Adolescents

**DOI:** 10.3389/fpsyg.2021.720199

**Published:** 2021-08-31

**Authors:** Jeremy T. Goldbach, Sheree M. Schrager, Mary Rose Mamey, Harmony Rhoades

**Affiliations:** ^1^Suzanne Dworak-Peck School of Social Work, University of Southern California, Los Angeles, CA, United States; ^2^Department of Graduate Studies and Research, California State University, Dominguez Hills, Carson, CA, United States

**Keywords:** sexual minority adolescents, minority stress theory, behavioral health, psychometrics, LGBT

## Abstract

**Objective:** Sexual minority adolescents (SMA) experience numerous behavioral health disparities, including depression, anxiety, substance use, non-suicidal self-injury, and suicidality. The primary framework to understand these disparities is minority stress theory, which frames this disproportionate burden as the result of discrimination, violence, and victimization in a homophobic culture. Empirical examinations of minority stress among SMA have been limited by lack of diverse samples or validated measures. This study engaged a national community sample of SMA to confirm reliability and validity of the Sexual Minority Adolescent Stress Inventory (SMASI).

**Method:** A national sample of 2,310 SMA aged 14–17 was recruited in the United States through a hybrid social media and respondent-driven sampling approach. Item response theory and confirmatory factor analysis established the psychometric properties of the SMASI in this sample; minority stress was modeled as a latent variable in several regression models to verify criterion and divergent validity.

**Results:** In this national sample (M age = 15.9; 64% female and 60% White), the factor structure of the SMASI and its 11 subscales was confirmed and shown to be invariant by demographic characteristics. Minority stress as measured by the SMASI was significantly associated with all mental and behavioral health outcomes.

**Conclusions:** This study provides evidence that SMASI is a reliable, valid, and important tool for better understanding minority stress and subsequent health and mental health consequences among SMA.

## Introduction

Numerous behavioral health disparities exist for sexual minority adolescents (SMA) when compared to their heterosexual peers. Adolescents who identify as a sexual minority are more likely to meet criteria for both internalizing and externalizing disorders than their heterosexual peers (Katz-Wise et al., 2015; Kaufman et al., 2020). Indeed, SMA report disparate rates of behavioral health concerns including depression, anxiety, and non-suicidal self-injury (NSSI; Hendricks and Testa, 2012; Luk et al., 2018), substance misuse (Goldbach et al., 2017a; Bränström and Pachankis, 2018), and suicide attempt (Di Giacomo et al., 2018; Raifman et al., 2020). Meta-analyses have found that SMA are almost 3 times more likely to report a history of suicidality and 5 times more likely to attempt suicide than their heterosexual peers (Marshal et al., 2011).

The primary framework for understanding the disparities found among sexual minorities is minority stress theory (Rosario et al., 2002; Meyer, 2003; Hatzenbuehler et al., 2009), which has been endorsed by the Centers for Disease Control Prevention (2011); National Academy of Medicine (2015), and Healthy People 2020 (2020). The theory suggests that discrimination, violence, and victimization (i.e., distal stressors) due to a pervasive homophobic culture are internalized (i.e., proximal stress) and the most probable driving mechanisms of mental health disparities among sexual minorities, including SMA (Meyer, 2003; Goldbach and Gibbs, 2017). For example, Hatzenbuehler et al. (2009) outlined a psychological mediation framework whereby sexual minorities are exposed to discriminatory experiences in the built environment; these experiences are internalized and cause elevation in emotion dysregulation, social and interpersonal problems, and cognitive processes that confer risk of psychopathology; and these processes lead to poorer behavioral health outcomes. In short, as Hatzenbuehler and Pachankis (2016) rightly noted in their review, stigma occurs at multiple levels for LGBT youth; disrupts cognitive, affective, interpersonal and physiological responses; and can likely only be addressed through multilevel frameworks.

Numerous cross-sectional studies have attributed health outcomes among adolescents to minority stressors including negative disclosure experiences with family and peers (Haas et al., 2010; McGeough and Sterzing, 2018; Poštuvan et al., 2019; Gamarel et al., 2020), homelessness (Rice and Barman-Adhikari, 2014; Tyler and Ray, 2019), perceived burdensomeness (Baams et al., 2018; Fulginiti et al., 2020) in-school victimization (bullying) by students and faculty members (Toomey et al., 2013; Norris and Orchowski, 2020), and experiences of violence (Kosciw et al., 2012; Sterzing et al., 2017; Schwab-Reese et al., 2021). Fear of rejection from family may also lead SMA to not disclose their identity to parents (Padilla et al., 2010), and lack of family support is often cited as a precursor to SMA homelessness (e.g., Ryan et al., 2009). Given that stigmatizing experiences can disrupt developmental tasks during adolescence and contribute to negative outcomes (Clatts et al., 2005), minority stress has understandably been a significant focus of etiological study in SMA health.

Prior work on minority stress and mental health has been fraught with significant methodological concerns. First, although national studies have established epidemiological differences in health for SMA when compared to their heterosexual peers (see, for example, Cochran et al., 2016), few national studies have examined the etiology of mental health concerns in this population. Some studies have examined minority stress and behavioral health in adolescents (such as those previously noted) but lack a diverse national sampling frame. Studies supported by national sampling strategies, on the other hand, have either focused on single-item assessments of discrimination (for example, bias-based bullying questions found in the Youth Risk Behavior Survey; Centers for Disease Control Prevention, 2019), been primarily focused on adults (e.g., Hatzenbuehler et al., 2017), or more recently, focused on generations of adults (e.g., older, younger; Meyer et al., 2020).

A second concern of the existing research on minority stress among SMA is a near exclusive reliance on poorly constructed measures of the phenomenon. Indeed, a review of psychometric measurements assessing discrimination against sexual minorities found that across 162 articles, nearly all had suboptimal psychometric properties and were intended for adults (Morrison et al., 2018). This is of critical importance, because valid and reliable measurement is a necessary antecedent to explanatory research, intervention efforts (Wolchik and Sandler, 2013), and clinical assessment (Watkins et al., 1995; Groth-Marnat, 2009). Further, the lack of operationalization of minority stress during adolescence is notable, given that this developmental time period is both critical to healthy development and includes uniquely stressful milieu such as living at home and being in school (Goldbach and Gibbs, 2017).

The first comprehensive measure of minority stress designed for use with adolescents, the Sexual Minority Adolescent Stress Inventory (SMASI), was recently developed (Schrager et al., 2018) and validated (Goldbach et al., 2017b) with a small sample of primarily single-state participants (*N* = 346). Consistent with minority stress theory, the measure includes subscales that represent both proximal and distal stressors. Emerging evidence suggests the SMASI may have utility for understanding behavioral health outcomes in both general population (e.g., Goldbach et al., 2017b) and clinical (e.g., Fulginiti et al., 2020) samples. However, the reliability and validity of this measure have not been established in a large national sample, and questions remain about the generalizability of previous findings to broader populations of sexual minority youth.

The study described here sought to address these barriers to research with SMA. We relied on a national purposive sampling framework with both direct (advertising) and indirect (respondent-driven sampling, or RDS) recruitment methods and representation from all 50 states and the District of Columbia. To our knowledge, this represents one of the largest national studies of minority stress and behavioral health among SMA in the United States to date. Second, we used the SMASI, a comprehensive measure of minority stress, to verify hypothesized relationships between minority stress and health outcomes. Recognizing the measure had yet to be validated in a diverse sample independent of its original development, we report on its utility as a measure of stress as well. Thus, we intended to make two contributions to future research and practice: (a) to conduct a confirmatory validation of the SMASI to establish its psychometric properties in a novel national community sample; and (b) to examine the relationship between minority stress and behavioral health outcomes, including depression, anxiety, suicidality, and substance use patterns, to inform intervention development.

## Method

### Participants and Procedures

This study involved a national sample of adolescents between 14 and 17 years old who were recruited for participation between May 2018 and April 2019 as part of a larger longitudinal study of sexual minority youth (Schrager et al., 2021). In brief, participants were recruited through a hybrid RDS (Heckathorn, 2011) approach, a type of snowball-sampling technique that allows participants in the study to recruit others in their network who may be hard to reach otherwise. Direct outreach efforts were made via advertising on Facebook, Instagram, and YouTube with ads targeted by age and interests. Ads varied slightly by platform but included language asking youth to “share your voice” and described basic details of the research study and incentives that participants could earn. Youth were eligible to participate if they were between 14 and 17 years old. Given the complexity of gender-related minority stress as separate from sexual minority stress in both theory and measurement (Testa et al., 2015), this study was restricted to youth who self-identified as cisgender male or female (i.e., assigned sex at birth that corresponded with current binary gender identity) and also identified as anything other than “100% heterosexual.”

The university institutional review board granted a waiver of parental consent to allow those younger than the age of consent (18 in most U.S. states) to participate. Given that obtaining consent may inadvertently “out” participants to their parents, which could result in negative consequences, it was determined that obtaining consent had the potential to increase harm to participants. Thus, participants who provided assent received the main survey and were then redirected to a separate Qualtrics survey page for incentive payment that asked for their private email address, where a $15 Amazon gift card was sent. For the RDS component, participants were asked whether they knew other sexual minority youth and if so, whether they would consider referring them to the study. Those who confirmed interest received three unique links to distribute to up to three friends. Each successful referral resulted in an additional $10 gift card to the recruiter.

A final baseline sample of 2,559 eligible adolescents was obtained. Because the present analysis was meant to confirm the findings of the original SMASI development in a national sample, and in the interest of not grouping participants with highly dissimilar sexual identities into a single “other” subgroup, we restricted the analytic sample to participants who identified as lesbian, gay, bisexual, pansexual, or queer. Consequently, 249 participants in the parent study who did not express one of these identities were excluded from the current analyses, resulting in an analytic sample of 2,310 sexual minority adolescents.

### Measures

#### Demographics

Demographic variables used to assess invariance included age, sexual identity, gender, race, region, and urbanicity. Sexual identity categories were created based on three items: sexual attraction [measured as mostly heterosexual (straight), bisexual or pansexual, mostly homosexual (gay or lesbian), 100% homosexual (gay or lesbian), or unsure]; gender identity; and an open-ended question asking youth to identify their sexual orientation. For analytic purposes, sexual identity was categorized as gay, lesbian, bisexual or pansexual, or queer. To be consistent with the population for which the SMASI was developed (Schrager et al., 2018), recognizing that minority stress is fundamentally a theory of stigmatized same-sex attracted identity, youth who identified as another sexual identity (e.g., “asexual”) or did not express a particular identity (e.g., “questioning”) were not included in the present analyses. As noted, only cisgender individuals were eligible for participation, which was coded as 0 (*female*) and 1 (*male*) for analysis. To assess race and ethnicity, participants were asked to select one of the following options: Native American, American Indian, or Alaska Native; Asian or Pacific Islander; Black or African American; White or Caucasian; Latino or Hispanic; multiracial; or race and ethnicity not listed. For invariance tests, race and ethnicity was grouped into White or Caucasian, Black or African American, Latino or Hispanic, and other. Because ZIP codes were asked of all potential respondents to determine whether they were currently residing in the United States, these were also recoded to identify participants' region and urbanicity. Participants were assigned to one of five regions (West, Southwest, Midwest, Southeast, and Northeast) based on the state in which their ZIP code was located. Urbanicity was derived from the ZIP code approximation for Rural-Urban Commuting Areas, version 3.1 (Cromartie, 2020). For analytic purposes, all ZIP codes were recoded into urban (RUCA codes 1, 1.1, 2.0, 2.1, 4.1, 5.1, 7.1, 8.1, and 10.1) or rural (RUCA codes 3.0, 4.0, 5.0, 6.0, 7.0, 7.2, 8.0, 8.2, 9.0, 10.0, 10.2, and 10.3). Work was assessed through a single question—“Are you currently working?”—with response options of “yes, full-time”; “yes, part-time”; “no, but I have previously had a job”; “no, and I have not previously had a job”; and “decline to answer.” Participants who reported current or past employment were presented with the 10 SMASI items that comprise the optional work subscale. School attendance was measured with a single question asking if the participant was currently enrolled in school (0 = *no* and 1 = *yes*).

#### Minority Stress

The SMASI (Schrager et al., 2018) is a comprehensive 64-item measure that includes 10 main subscales composed of 54 items that can be answered by all sexual minority youth (e.g., “I have heard a family member make negative comments about LGBTQ people”; “Other students make fun of me for being LGBTQ”) and an optional work subscale with 10 additional items assessing experiences at work among adolescents who have ever been employed (e.g., “My workplace does not protect LGBTQ employees”). Subscales of the main 54-item measure include social marginalization (8 items); family rejection (11 items); internalized homonegativity (7 items); identity management (3 items); homonegative climate (4 items); intersectionality (3 items); negative disclosure experiences (5 items); religion (5 items); negative expectancies (3 items); and homonegative communication (5 items). All items are framed as binary indicators of whether a respondent experienced the corresponding minority stressor in their lifetime, and youth are asked to indicate whether they ever experienced each stressor (1 = *yes*, 0 = *no*) ever experienced each stressor. Positive responses trigger a follow-up binary item assessing whether that experience has happened in the past 30 days (1 = *yes*, 0 = *no*). The overall SMASI can be operationalized for analysis as either a sum score (theoretical range = 0–54) or a latent construct with the 10 main subscales as manifest variables. The subscale scores are calculated as percentages reflecting the number of endorsed items in the subscale, with a theoretical range between 0 and 100. This allows for a more accurate calculation of scores, standardizes the comparisons of subscales with different number of items, and accounts for any missing data. In accordance with the published scoring rules, idiographic mean substitution was used to replace any missing values for calculation of manifest total score only. As in previous work, psychometric analyses focused on the lifetime measure, given a high degree of expected variability between stressors in a 30-day time frame (Goldbach et al., 2017b; Schrager et al., 2018).

#### Depression

The CES-D-4 (Melchior et al., 1993; α = 0.85) is a four-item scale used to measure past-week depressive symptoms, which include feelings of depression, loneliness, sadness, and crying spells. Response options range from 0 [*rarely or none of the time* (<1 day)] to 3 [*most or all of the time* (5–7 days)]. Scores are summed (theoretical range = 0–12), with higher values indicating more depressive symptoms.

#### Anxiety

The GAD-7 (Mossman et al., 2017; α = 0.90) was used to measure anxiety. The scale features seven items that ask how often a person has been bothered by problems during the past 2 weeks. Response options are based on a 4-point Likert-type scale: 0 = *not at all*, 1 = *several days*, 2 = *more than half the days*, and 3 = *nearly every day*. Scores are calculated by summing the scores of each item, with a theoretical range between 0 and 21.

#### Post-traumatic Stress Disorder

PTSD was measured with the abbreviated PCL-C (Lang et al., 2012; α = 0.85), a 6-item PTSD checklist used to understand problems and complaints related to stressful life experiences. Respondents are asked to answer questions based on the past month, with response options ranging between 1 (*not at all*) to 5 (*extremely*). The range of total sum scores is between 5 and 30.

#### Suicidality

Four questions pertained to suicidality based on feelings and behaviors in the past 12 months were taken from the Youth Risk Behavior Survey (Centers for Disease Control Prevention, 2010) and scored according to recommendations by Brener et al. (2002). Suicidal ideation (“Did you ever seriously consider attempting suicide?”), plan (“Did you make a plan about how you would attempt suicide?”), and suicide attempt (“How many times did you actually attempt suicide?”) were programmed with dichotomous response options (1 = *yes* and 0 = *no*). NSSI (“How many times did you do something to purposely hurt yourself without wanting to die, such as cutting or burning yourself on purpose?”) was dichotomized (1 = *at least one incident*, 0 = *no incidents*).

#### Substance Use

Similarly, we used the Youth Risk Behavior Survey (Centers for Disease Control Prevention, 2010) substance use subscale for lifetime and past-30-day use of alcohol, tobacco, marijuana, prescription pain relievers, prescription tranquilizers, and prescription stimulants without a doctor's note. Prescription pain relievers, tranquilizers, and stimulants were combined into a single outcome of prescription drug use. All items had dichotomous response options (yes or no). Affirmative responses to lifetime use triggered the past-30-day use question.

#### General Stress

The Perceived Stress Scale (PSS: Cohen et al., 1983; α = 0.82) is composed of 10 items that measure how often a person has experienced a stressful situation during the past 30 days. Likert-type response options range between 0 (*never*) to 4 (*very often*) and are summed to provide a total score (theoretical range = 0–40).

### Analytic Approach

Descriptive statistics or frequencies of demographic information, all SMASI items, and health outcomes were first examined. To address the goal of establishing the SMASI's psychometric properties in a national community sample, we conducted a confirmatory factor analysis to verify the structure of each of the 11 subscales by examining the global fit [the evaluation of overall fit of the model, such as chi-square, comparative fit index (CFI), and root mean square error of approximation (RMSEA)] and localized fit (the evaluation of standardized loadings). We subsequently replicated the Rasch model under item response theory (IRT) analysis previously used to develop the measure. Discrimination and difficulty parameters for the overall SMASI scale and the 11 subscales were assessed using this IRT framework, allowing for a more specific evaluation of how items operate in comparison to each other and in the context of the larger scale. Models were run to verify both the configural and scalar variance for measurement invariance for the scale and 11 subscales across sexual identity, age, gender, region, urbanicity, and racial and ethnic groups. Finally, we calculated omega (ω) coefficients to assess the internal consistency of the whole scale and each subscale.

To address the second goal of examining the relationship between minority stress and health outcomes, two competing factor structures of the models were assessed. The first modeled minority stress as a single latent variable, with the 10 main SMASI subscales modeled as continuous, manifest variables, replicating the prior published model (Goldbach et al., 2017b). The second modeled minority stress as a second-order latent variable with proximal and distal as first-order latent variables and the 10 main SMASI subscales modeled again as continuous manifest variables, each of which loaded onto either the proximal (internalized homonegativity, identity management, and negative expectances) or distal (negative disclosure experiences, family rejection, homonegative communication, homonegative climate, social marginalization, intersectionality, and religion) latent variable. Global fit and localized fit were assessed for all models, and modification parameters were used to inform added pathways (correlations) to better improve model fit. Criterion validity was tested by regressing depression, anxiety, PTSD, suicidality, and substance use onto the latent minority stress construct. Standardized betas (β) were used to report on continuous outcome variables, and odds ratios (ORs) were used to report on dichotomous outcome variables. Divergent validity was assessed by determining whether the SMASI maintained statistical significance after controlling for general stress. Statistical analyses were conducted using SPSS v. 25 and Mplus v. 8.4. To address inflated family-wise error, all *p*-values of significance tests were adjusted for type I error using the procedure developed by Benjamini and Hochberg (1995).

## Results

Analyses included 2,310 adolescent sexual minority participants. Both the small percentage (17.7%) of participants who were recruited through RDS and the short chains resulting from this recruitment effort precluded weighting the sample by recruitment chain. However, RDS-derived participants did not materially differ in outcomes from direct referrals. Descriptive statistics and frequencies of the demographic information are shown in Table 1. Participants had a mean age of 15.91 years (*SD* = 0.97). Similar to the U.S. census (U. S. Census Bureau, 2012), most participants were from urban areas (*n* = 1,857; 80.4%), female (*n* = 1,466; 63.5%), and White or Caucasian (*n* = 1,386; 60.0%). Participants were most likely to identify as bisexual or pansexual (*n* = 1,167; 50.5%), followed by gay (*n* = 618; 26.8%), lesbian (*n* = 440; 19.0%), and queer (*n* = 85; 3.7%). Participants were distributed across U.S. regions, with most participants living in the West (*n* = 559; 24.2%), Southeast (*n* = 540; 23.4%), or Northeast (*n* = 497; 21.5%), and fewer in the Midwest (*n* = 412; 17.8%) and Southwest (*n* = 302; 13.1%).

**Table 1:** Demographics of participating youth (*N* = 2,311).

**Demographics**	***n* (%)**
Gender	
Male	844 (36.5)
Female	1,466 (63.5)
Age	
14	229 (9.9)
15	509 (22.0)
16	804 (34.8)
17	768 (33.2)
Race and ethnicity	
Native American, American Indian, or Alaska Native	53 (2.3)
Asian or Pacific Islander	148 (6.4)
Black or African American	178 (7.7)
White or Caucasian	1,386 (60.0)
Latino or Hispanic	345 (14.9)
Multiracial	199 (8.6)
Sexual orientation	
Gay	618 (26.8)
Lesbian	440 (19.0)
Bisexual or pansexual	1,167 (50.5)
Queer	85 (3.7)
Region	
West	559 (24.2)
Southwest	302 (13.1)
Midwest	412 (17.8)
Southeast	540 (23.4)
Northeast	497 (21.5)
Urbanicity	
Rural	453 (19.6)
Urban	1,857 (80.4)

Frequencies of all 64 individual SMASI items were assessed to confirm that no single item presented uneven cell sizes between endorsed (“yes”) and not endorsed (“no”) responses that could present problems in later analyses. Because sufficient distributions were observed for each item, discrimination and difficulty parameters under IRT were used to assess all 64 SMASI items using the two-parameter logistic model. Difficulty parameters—the level of underlying traits a person needs to have to endorse the item (“yes”)—produced values between −2.61 and 3.69 standard deviations from the mean. The usual range falls between −2.00 and 2.00; our results indicate that it takes an average amount of the underlying experience described in the item to endorse that item. Discrimination parameters—values used to differentiate between those who will or will not endorse the item—had an average range of 0.31 and 1.27, with the standard range between −0.50 and 2.50. Subscales also demonstrated acceptable parameter ranges for difficulty and discrimination. Confirmatory factor analyses for the 11 subscales of the SMASI were examined to understand factor structure and unidimensionality by assessing global and localized fit. All subscales had acceptable global fit (CFI > 0.93; RMSEA < 0.09), and no localized ill fit.

### Invariance Testing

Invariance tests were conducted to assess whether our data demonstrated configural and scalar invariance across groups. Configural invariance (non-restrictive model) was assessed by examining the CFI (≥0.90) and considering the RMSEA for global fit. Scalar invariance (restrictive model) was assessed by constraining the loading and thresholds equal across groups and examining the change in CFI (ΔCFI). A decrease of more than 0.01 would result in the examination of other parameters, including loadings and thresholds, and modification indexes.

Prior to assessing invariance across groups for each subscale, measurement invariance was tested for the whole scale across gender, age, race and ethnicity, urbanicity, region, and sexual orientation. Each test of invariance demonstrated no decrement in fit as models were constrained from equal form (configural invariance) to equal loadings and thresholds (scalar invariance) through evaluation of the CFI. Change in CFI did not exceed a decrease of 0.01, suggesting that no systematic differences emerged among groups for the whole scale.

#### Gender

Gender (male vs. female) showed configural invariance in all subscales (CFI ≥ 0.910) and scalar invariance in seven subscales. Subscales of identity management (ΔCFI = −0.128), homonegative communication (ΔCFI = −0.054), homonegative climate (ΔCFI = −0.014), and intersectionality (ΔCFI = −0.014) showed a decrease > 0.01. The examination of parameters both with and without constraints and overall global fit showed adequate fit between the models and the observed data.

#### Age

Age, categorized as 14, 15, 16, and 17, showed configural invariance in each subscale (CFI ≥ 0.935) and scalar invariance in all but the work subscale (ΔCFI = −0.014). A further examination of this subscale's parameters showed good global fit (CFI = 0.983; RMSEA = 0.042) and no localized ill fit (loadings ≥ 0.792).

#### Race and Ethnicity

Race was assessed by examining White or Caucasian, Black or African American, Hispanic or Latino, and other categories. Each subscale had configural invariance (CFI ≥ 0.899). Negative disclosures, family rejection, internalized homonegativity, social marginalization, and religion subscales also demonstrated scalar invariance (CFI = 0.942–0.983). Identity management (ΔCFI = −0.016), negative expectancies (ΔCFI = −0.020), homonegative communication (ΔCFI = −0.025), and homonegative climate (ΔCFI = −0.034) subscales showed decreases in CFI > 0.01.

The intersectionality subscale (ΔCFI = −0.588) was examined more closely, because a single item (“As an LGBTQ person in my racial/ethnic community, I feel like I am a minority within a minority”) had a threshold and loading in the White or Caucasian group that differed in direction and magnitude from the other three groups, resulting in a decrement in fit. In the Black or African American, Hispanic or Latino, and other groups (i.e., racial and ethnic minorities), intersectionality demonstrated scalar invariance (CFI = 0.987).

Additionally, the work subscale would not converge because a single item in the Black or African American group (“I have been physically assaulted by people at my work because I am LGBTQ”), had no endorsed (yes) responses. When this question was removed, the work subscale demonstrated scalar invariance across all four racial and ethnic groups (CFI = 0.983). To examine the complete subscale, all 10 items were tested among the three groups with response variability (White or Caucasian, Hispanic or Latino, and other), and again, the work subscale demonstrated scalar invariance (CFI = 0.978).

#### Urbanicity

Configural invariance was also found for all subscales when comparing urban and rural adolescents (CFI ≥ 0.927). Seven subscales demonstrated scalar invariance: Identity management (ΔCFI = −0.013), negative expectancies (ΔCFI = −0.020), homonegative climate (ΔCFI = −0.029), and intersectionality (ΔCFI = −0.026) had a decrement in fit between models, although the constrained models of each subscale demonstrated good global fit (CFI = 0.954–0.985).

#### Region

Region had configural invariance for all subscales (CFI ≥ 0.939) and demonstrated scalar invariance for all subscales except identity management (ΔCFI = −0.013) and intersectionality (ΔCFI = −0.024). However, the global fit for both was adequate (CFI = 0.985 and 0.946; RMSEA = 0.040 and 0.102, respectively).

#### Sexual Orientation

Sexual orientation was evaluated across participants who identified as gay, lesbian, bisexual or pansexual, and queer. All subscales had configural invariance. Subscales of identity management (ΔCFI = −0.013), negative expectancies (ΔCFI = −0.020), homonegative communication (ΔCFI = −0.028), homonegative climate (ΔCFI = −0.034), and intersectionality (ΔCFI = −0.591) all showed decrement in fit, and all but intersectionality showed acceptable global fit.

### Summary of SMASI Psychometric Properties

The overall SMASI had a mean of *M* = 20.32 (*SD* = 9.91), indicating that adolescents in our study reported an average of 20 minority stress experiences during their lifetime at the time they took the survey. Overall, reliability for the SMASI whole scale was excellent (ω = 0.97), and its subscales showed good to excellent reliability as well (ω = 0.76–0.96). Means, standard deviations, and reliabilities for the scale and subscales are presented in Table 2. Correlations among the SMASI subscales (Table 3) were all statistically significant at the *p* < 0.001 level with small to moderate coefficients, consistent with the assertion that the subscales represent different aspects or domains of the larger minority stress construct.

**Table 2:** Means, standard deviations, and reliabilities (ω) of SMASI total score and subscale scores.

**Scale**	**Items**	***M* (*SD*)**	**ω**
SMASI total score	54	20.32 (9.91)	0.965
F1: Identity management	3	44.91 (36.52)	0.774
F2: Negative expectancies	3	53.23 (37.08)	0.813
F3: Negative disclosure experiences	5	38.84 (30.14)	0.770
F4: Family rejection	11	42.28 (30.63)	0.943
F5: Internalized homonegativity	7	26.37 (29.68)	0.936
F6: Homonegative communication	5	76.46 (24.64)	0.762
F7: Homonegative climate	4	41.34 (35.61)	0.884
F8: Social marginalization	8	12.71 (19.63)	0.928
F9: Intersectionality	3	32.52 (36.00)	0.833
F10: Religion	5	30.27 (27.73)	0.930
F11: Work	10	10.22 (18.94)	0.957

**Table 3:** Correlations among SMASI subscales.

	**1**	**2**	**3**	**4**	**5**	**6**	**7**	**8**	**9**	**10**
1: Identity management										
2: Negative expectancies	0.375									
3: Negative disclosure experiences	0.138	0.240								
4: Family rejection	0.236	0.403	0.385							
5: Internalized homonegativity	0.463	0.433	0.199	0.318						
6: Homonegative communication	0.256	0.364	0.309	0.384	0.256					
7: Homonegative climate	0.187	0.357	0.290	0.192	0.255	0.364				
8: Social marginalization	0.133	0.295	0.334	0.290	0.205	0.288	0.514			
9: Intersectionality	0.177	0.281	0.196	0.398	0.244	0.355	0.224	0.343		
10:Religion	0.182	0.294	0.280	0.566	0.268	0.305	0.227	0.307	0.328	
11: Work^a^	0.138	0.275	0.273	0.221	0.194	0.226	0.361	0.519	0.223	0.199

*p < 0.001 for all correlations*.

a *Participants who received Work subscale: N = 1,125*.

### Criterion Validity

Minority stress was next modeled as a latent construct, wherein the 10 primary SMASI subscales (omitting the optional work subscale) were modeled as continuous, manifest variables. In its first iteration, each manifest variable loaded onto the latent construct of the SMASI and provided poor model fit [$\chi_{\left(35\right)}^{2}$ = 1,255.02, *p* < 0.001; CFI = 0.778; RMSEA = 0.123; SRMR = 0.066]. Correlations informed by the modification indexes were incorporated one at a time, starting with largest value, until good global and localized fit were achieved. The final model consisted of nine correlations among the manifest variables to provide good global fit [$\chi_{\left(26\right)}^{2}$ = 144.09, *p* < 0.001; CFI = 0.978; RMSEA = 0.044; SRMR = 0.022]: homonegative climate with social marginalization, intersectionality, religion, and family rejection; family rejection with religion and homonegative climate; internalized homonegativity with identity management and negative expectancies; and identity management with negative expectancies.

A competing model was tested that placed SMASI as a second-order latent construct and proximal and distal constructs as first-order factors. Each of the 10 primary SMASI subscales were modeled as continuous, manifest variables, with each loading onto its corresponding proximal or distal factor. This model provided poor global fit: $\chi_{\left(35\right)}^{2}$ = 1,247.84, *p* < 0.001; CFI = 0.779; RMSEA = 0.122; SRMR = 0.066. Modification indexes again were used to inform appropriate correlations among manifest variables; only those variables in the proximal or distal factor were allowed to correlate. This final model also incorporated nine correlations to provide good global fit: $\chi_{\left(26\right)}^{2}$ = 153.66, *p* < 0.001; CFI = 0.977; RMSEA = 0.046; SRMR = 0.023.

Because the second-order factor structure did not substantially improve model fit over the original model with a single latent variable, the latter was retained in the structural analyses used to assess criterion validity. Tables describing the bivariate correlations between the SMASI lifetime variables (whole scale and each subscale) and the 15 behavioral health outcomes are provided as Supplementary Material.

#### Mental Health Outcomes

Minority stress was significantly and positively associated with symptoms of depression (β = 0.37, *p* < 0.001), anxiety (β = 0.40, *p* < 0.001), and PTSD (β = 0.48, *p* < 0.001), indicating that youth who reported more extensive minority stress experiences also reported significantly worse mental health. Minority stress was also significantly related to a history of suicidal ideation (*OR* = 2.10; *p* < 0.001), suicide plan (*OR* = 2.10; *p* < 0.001), suicide attempt (*OR* = 1.97; *p* < 0.001), and NSSI (*OR* = 1.73; *p* < 0.001). Findings indicate that minority stress is associated with an increased risk of each indicator of suicidality and NSSI.

#### Substance Use

Greater minority stress was significantly associated with increased lifetime use of alcohol (*OR* = 1.37; *p* < 0.001), marijuana (*OR* = 1.34; *p* < 0.001), tobacco (*OR* = 1.46; *p* < 0.001), and prescription drugs (*OR* = 1.62; *p* < 0.001). Similarly, minority stress was also associated with past-30-day use of each substance: alcohol (*OR* = 1.47; *p* < 0.001), marijuana (*OR* = 1.33; *p* < 0.001), tobacco (*OR* = 1.51; *p* < 0.001), and prescription drugs (*OR* = 1.82; *p* < 0.001).

### Divergent Validity

Finally, we examined the PSS to understand its relationship with the SMASI and whether the SMASI continues to contribute to the variance accounted for in the health outcome models after controlling for this measure of general stress. The PSS was modeled using the total score as a manifest variable. A moderate, significant correlation emerged between the total PSS sum score and the total SMASI sum score (*r* = 0.37, *p* < 0.001). A moderate, significant correlation also emerged when SMASI was instead modeled as a latent construct (*r* = 0.43, *p* < 0.001).

After controlling for the PSS in regression models, the SMASI maintained its statistically significant associations with all health outcomes. Table 4 reports the unadjusted and adjusted betas and odds ratios corresponding to the association of the latent SMASI variable and each behavioral health outcome.

**Table 4:** Health outcomes and associations with minority stress (SMASI), with and without adjusting for general stress (PSS).

**Outcome**	***M*** **(*SD*)**	**SMASI beta** **(unadjusted)**	**SMASI beta** **(adjusted)**
Depression	6.31 (3.40)	0.37	0.12
Anxiety	11.77 (5.95)	0.40	0.13
PTSD	17.42 (6.02)	0.48	0.24
	***n*** **(%)**	**SMASI OR** **(unadjusted)**	**SMASI OR** **(adjusted)**
Suicidality: ideation	1,005 (43.5)	2.10	1.59
Suicidality: plan	660 (28.6)	2.10	1.68
Suicidality: attempt	383 (16.6)	1.97	1.65
Non-suicidal self-injury	995 (43.1)	1.73	1.34
Lifetime alcohol	1,283 (55.5)	1.37	1.30
Lifetime marijuana	796 (34.4)	1.34	1.27
Lifetime prescription drugs	393 (17.0)	1.62	1.40
Lifetime tobacco	671 (29.0)	1.46	1.38
30-day alcohol	641 (27.7)	1.47	1.40
30-day marijuana	438 (18.9)	1.33	1.24
30-day prescription drugs	146 (6.3)	1.82	1.50
30-day tobacco	363 (15.7)	1.51	1.40

*p < 0.001 for all coefficients*.

## Discussion

To our knowledge, this is one of the largest national studies of minority stress and health among adolescents (aged 14–17) in the United States with data across all states, a rural sample reflective of the greater U.S. census, and a reasonable balance of participants based on cisgender male and female status and racial and ethnic identity. As described in earlier papers on the development of the SMASI, it has been nearly 20 years since the original minority stress theory was formally described by Meyer (2003) and further refined by Hatzenbuehler et al. (2009). However, despite thousands of studies attributing behavioral health patterns to minority stress, our inability to effectively measure this theorized construct has remained a consistent challenge to both etiological research and intervention development. To address these concerns, our study had two primary objectives. First, we sought to conduct a confirmatory analysis of the SMASI to establish its psychometric properties in a national sample. Second, we intended to validate the relationships between minority stress (as measured by the 11 domains of the SMASI) and markers of behavioral health including depression, anxiety, suicidality, and substance use patterns.

To the first point, the SMASI has been tested through prior exploratory research, although the much smaller samples used in the original developmental studies (Goldbach et al., 2017b; Schrager et al., 2018) were heavily skewed toward urban, West Coast youth. Furthermore, the same underlying sample was used to develop a measure with good psychometric properties and establish its preliminary criterion and divergent validity. As such, the present study marks the first true validation of the SMASI in a new sample that included youth from all 50 states and the District of Columbia. Results from this study show that the SMASI and its subscales remain highly reliable, and invariance tests confirmed that it is appropriate to use the SMASI with members of diverse subpopulations of sexual minority youth. Importantly, the larger sample allowed us to extend previous invariance results by (a) including a subgroup of youth who identified as queer and (b) establishing the measure's invariance by urbanicity and U.S. region. Given that sexual minorities in the United States who live in rural areas tend to report higher rates of discrimination, unequal access to competent health care, and poorer behavioral health (e.g., Willging et al., 2006; Fisher et al., 2014), it was important to establish that the SMASI can be applied across the United States without potentially biasing findings due to measurement variance.

Regarding behavioral health, our data provide further evidence for the study of minority stress as a driving mechanism for behavioral health patterns among adolescents. Similar to the existing literature, reporting higher levels of minority stress was associated with negative internalizing (including depression, anxiety, PTSD, and suicidal ideation and planning) and externalizing (suicide attempt and substance use) symptomology. One novel contribution of our study is its demonstration of an association between minority stress and NSSI. Although prior research has documented increased rates of NSSI among sexual minorities (e.g., Batejan et al., 2015) and general risk and protective factors for SMA in school-based studies (e.g., Taliaferro and Muehlenkamp, 2017), studies of minority stress and NSSI remain sparse. Despite some encouraging studies being conducted among college students (Muehlenkamp et al., 2015), we assert that studies of minority stress and NSSI may consider using the SMASI in future inquiries.

An additional benefit of relying on a comprehensive measure of minority stress such as the SMASI is our capacity to provide nuanced information about 11 discrete domains of minority stress that interact but remain independent. Although a full exploration of the unique relationships between each domain of stress and the various associated health outcomes is beyond the scope of the present paper (see Supplemental Material), the ability to conduct analyses at a level that goes beyond minority stress or even distal vs. proximal factors has the potential for endless inquiries. How might experiences of internalized homonegativity mediate the relation between family rejection and mental health? As youth age out of adolescence and into adulthood, how might changes in their reporting of identity management and social isolation change? Does it really “get better,” or do youth simply become better equipped to manage their stress? Understanding how minority stressors evolve throughout adolescence could lead toward the development of more targeted interventions that support youth during the many “critical windows” (Marín, 2016) of early life.

A final contribution of our study is the inclusion of our divergent validity analyses, which replicated a previous finding that the relationship between minority stress and health remains robust even after accounting for a measure of general stress. Given that a common critique of minority-related stress theories is that “stress is bad for everyone” regardless of minority status, we recognized the importance of interrogating our assumptions about minority stress in this national sample. As in our prior study (Goldbach et al., 2017b), reporting of minority stress remained strongly associated with all outcomes of interest, suggesting again that interventions that can reduce the presence of minority stress in the lives of SMA and help them better cope with these stressors are likely to be efficacious in addressing behavioral health concerns.

To that end, we believe that a more nuanced understanding of the minority stress and health relationship is necessary for intervention studies to move forward, and we hope that this paper forms a foundation for such work. As Meyer and Bayer (2013) stated in their review, there are currently “no determinative studies, such as randomized control trials, of the efficacy and effectiveness of school-based interventions [for SMA]” (p. 1767). Similarly, Hatzenbuehler and Pachankis (2016) suggest that addressing stigma among LGBT youth will require both clinical and public health intervention and that multicomponent interventions are likely to be the most effective.

Some interventions have emerged for LGB adults that build on a minority stress framework in the interpersonal setting. For example, Pachankis et al. (2015) developed an LGB-affirmative cognitive behavioral therapy for young adult gay and bisexual men with encouraging improvement for numerous behavioral health outcomes. However, less evidence exists to support adolescents. One intervention with substantial quasi-experimental findings does exist: the Family Acceptance Project (Ryan, 2010). However, this program is largely contingent on family participation. Unfortunately, because many SMA do not disclose their sexual identity to their family for fear of rejection, and lack of family support is common (Ryan et al., 2009; Padilla et al., 2010; Durso and Gates, 2012), this intervention has limited utility.

Other programs such as Affirmative Supportive Safe and Empowering Talk (Craig, 2013; Craig et al., 2014) have also found preliminary support through open pilot testing, focusing on building resilience, but their efficacy has yet to be established in the literature. Indeed, recent work to explore coping and supportive resources that can counteract the influence of minority stress including peer and family support, LGBT friendships, safety and access to resources suggest new veins for intervention (e.g., Goldbach and Gibbs, 2015; Salvati et al., 2018; Petrocchi et al., 2020; Weeks et al., 2020). Recent psychometric studies have also explored the importance of positive identity development in LGB people (Baiocco et al., 2018). Some naturalistic studies have also found that protective school climates (including those with gender and sexuality alliances) are associated with better mental health patterns, including lower suicidal ideation among adolescents (Hatzenbuehler et al., 2014). However, much remains to be explored in the area of broader systems and social climate. Indeed, interventions that do not require family involvement, have a strong theoretical and empirical foundation, and build on clearly defined mechanisms of change at both individual and structural levels (e.g., NIH Intervention Stage Model for Behavioral Intervention Research; Onken et al., 2014) are needed.

As always, our study findings should be considered with limitations in mind. Although the racial and ethnic (including multiracial) and rural–urban composition of this sample closely mirrored the U.S. population (Parker et al., 2015), participants were not a random, representative sample of SMA in the United States. Although we believe our hybrid RDS approach improved representation of young people who don't social media, we may have excluded adolescents without internet access or social media use. Related, although we included extensive protections for participant confidentiality, we recognize that some adolescents may not have felt comfortable participating in a study about LGBTQ topics because they have not disclosed their sexual identity to their family or others in their lives. Finally, because this was a cross-sectional study, understanding the temporal relationship between stigma (as measured by the SMASI) and health is not possible. Future studies should explore the temporal relationship between these two critical factors to the lives of SMA.

Despite these limitations, this study contributes to our understanding of the role of minority stress in the lives of SMA. These findings can help address some of the methodological concerns present in previous research on minority stress by providing evidence that the SMASI has excellent reliability in a diverse, national sample, and is strongly—and uniquely—associated with mental health, suicidality, and substance use outcomes. As such, the SMASI represents a reliable, valid, and important tool for research to better understand and hopefully ameliorate minority stress and subsequent health and mental health consequences among SMA.

## Data Availability Statement

Deidentified data supporting the conclusions of this article can be made available by the authors upon request and with appropriate IRB approval.

## Ethics Statement

The studies involving human participants were reviewed and approved by University of Southern California Social-Behavioral IRB. Written informed consent from the participants' legal guardian/next of kin was not required to participate in this study in accordance with the national legislation and the institutional requirements.

## Author Contributions

JG and SS conceptualized the study. SS and MM conducted all analyses and writing of methods and results. JG and HR were responsible for literature review and discussion. All authors reviewed the final version before submission.

## Conflict of Interest

The authors declare that the research was conducted in the absence of any commercial or financial relationships that could be construed as a potential conflict of interest.

## Publisher's Note

All claims expressed in this article are solely those of the authors and do not necessarily represent those of their affiliated organizations, or those of the publisher, the editors and the reviewers. Any product that may be evaluated in this article, or claim that may be made by its manufacturer, is not guaranteed or endorsed by the publisher.

## References

[B1] Baams L. Dubas J. S. Russell S. T. Buikema R. L. van Aken M. A. (2018). Minority stress, perceived burdensomeness, and depressive symptoms among sexual minority youth. J. Adolesc. 66, 9–18. 10.1016/j.adolescence.2018.03.015 29723686 PMC6919272

[B2] Baiocco R. Salvati M. Carone N. Ioverno S. Nappa M. R. Pistella J. (2018). Positive identity in sexual minorities: a contribution to the Italian validation of the Lesbian, Gay, and Bisexual Positive Identity Measure (LGB-PIM). Giornale Italiano di Psicologia 45, 953–978. 10.1037/t74138-000

[B3] Batejan K. L. Jarvi S. M. Swenson L. P. (2015). Sexual orientation and non-suicidal self-injury: a meta-analytic review. Arch. Suicide Res. 19, 131–150. 10.1080/13811118.2014.957450 25297459

[B4] Benjamini Y. Hochberg Y. (1995). Controlling the false discovery rate: a practical and powerful approach to multiple testing. J. R. Stat. Soc. Series B 57, 289–300. 10.1111/j.2517-6161.1995.tb02031.x

[B5] Bränström R. Pachankis J. E. (2018). Sexual orientation disparities in the co-occurrence of substance use and psychological distress: a national population-based study (2008–2015). Soc. Psychiatry Psychiatr. Epidemiol. 53, 403–412. 10.1007/s00127-018-1491-4 29450600 PMC5862943

[B6] Brener N. D. Kann L. McManus T. Kinchen S. A. Sundberg E. C. Ross J. G. (2002). Reliability of the 1999 youth risk behavior survey questionnaire. J. Adoles. Health 31, 336–342. 10.1016/S1054-139X(02)00339-7 12359379

[B7] Centers for Disease Control and Prevention (2010). Youth Risk Behavior Survey. Available online at: https://www.cdc.gov/yrbs (accessed June 2021).

[B8] Centers for Disease Control and Prevention (2011). Lesbian, Gay, Bisexual, and Transgender Health: Youth. Available online at: http://www.cdc.gov/lgbthealth/youth.htm (accessed June 2021).

[B9] Centers for Disease Control and Prevention (2019). Youth Risk Behavior Survey Questionnaire. Available online at: https://www.cdc.gov/yrbs (accessed June 2021).

[B10] Clatts M. C. Goldsamt L. Yi H. Gwadz M. V. (2005). Homelessness and drug abuse among young men who have sex with men in New York City: a preliminary epidemiological trajectory. J. Adolesc. 28, 201–214. 10.1016/j.adolescence.2005.02.003 15878043 PMC2755549

[B11] Cochran S. D. Björkenstam C. Mays V. M. (2016). Sexual orientation and all-cause mortality among US adults aged 18 to 59 years, 2001–2011. Am. J. Public Health 106, 918–920. 10.2105/AJPH.2016.303052 26985610 PMC4985116

[B12] Cohen S. Kamarck T. Mermelstein R. (1983). A global measure of perceived stress. J. Health Soc. Behav. 24, 386–396. 10.2307/2136404 6668417

[B13] Craig S. L. (2013). Affirmative Supportive Safe and Empowering Talk (ASSET): leveraging the strengths and resiliencies of sexual minority youth in school-based groups. J. LGBT Issues Couns. 7, 372–386. 10.1080/15538605.2013.839342

[B14] Craig S. L. Austin A. McInroy L. B. (2014). School-based groups to support multiethnic sexual minority youth resiliency: preliminary effectiveness. Child Adolesc. Soc. Work J. 31, 87–106. 10.1007/s10560-013-0311-7

[B15] Cromartie J. (2020). The Rural-Urban Commuting Area Codes ZIP Code File. Available online at: https://www.ers.usda.gov/data-products/rural-urban-commuting-area-codes.aspx (accessed June 2021).

[B16] Di Giacomo E. Krausz M. Colmegna F. Aspesi F. Clerici M. (2018). Estimating the risk of attempted suicide among sexual minority youths: a systematic review and meta-analysis. JAMA Pediatr. 172, 1145–1152. 10.1001/jamapediatrics.2018.2731 30304350 PMC6583682

[B17] Durso L. E. Gates G. J. (2012). Serving Our Youth: Findings From a National Survey of Services Providers Working With Lesbian, Gay, Bisexual and Transgender Youth Who Are Homeless or at Risk of Becoming Homeless. Los Angeles: The Williams Institute with True Colors Fund and The Palette Fund.

[B18] Fisher C. M. Irwin J. A. Coleman J. D. (2014). LGBT health in the midlands: a rural/urban comparison of basic health indicators. J. Homosex. 61, 1062–1090. 10.1080/00918369.2014.872487 24344731

[B19] Fulginiti A. Goldbach J. T. Mamey M. R. Rusow J. Srivastava A. Rhoades H.. (2020). Integrating minority stress theory and the interpersonal theory of suicide among sexual minority youth who engage crisis services. Suicide Life Threat. Behav. 50, 601–616. 10.1111/sltb.12623 32048340

[B20] Gamarel K. E. Watson R. J. Mouzoon R. Wheldon C. W. Fish J. N. Fleischer N. L. (2020). Family rejection and cigarette smoking among sexual and gender minority adolescents in the USA. Int. J. Behav. Med. 27, 179–187. 10.1007/s12529-019-09846-8 31925674 PMC7124998

[B21] Goldbach J. T. Gibbs J. (2015). Strategies employed by sexual minority adolescents to cope with minority stress. Psychol. Sex. Orient. Gender Divers. 2, 297–306. 10.1037/sgd0000124 26634221 PMC4663988

[B22] Goldbach J. T. Gibbs J. J. (2017). A developmentally informed adaptation of minority stress for sexual minority adolescents. J. Adolesc. 55, 36–50. 10.1016/j.adolescence.2016.12.007 28033502 PMC5272836

[B23] Goldbach J. T. Mereish E. H. Burgess C. (2017a). Sexual orientation disparities in the use of emerging drugs. Subst. Use Misuse 52, 265–271. 10.1080/10826084.2016.1223691 27759480

[B24] Goldbach J. T. Schrager S. M. Mamey M. R. (2017b). Criterion and divergent validity of the sexual minority adolescent stress inventory. Front. Psychol. 8:2057. 10.3389/fpsyg.2017.02057 29234292 PMC5712417

[B25] Groth-Marnat G. (2009). The five assessment issues you meet when you go to heaven. J. Pers. Assess. 91, 303–310. 10.1080/00223890902935662 20017059

[B26] Haas A. P. Eliason M. Mays V. M. Mathy R. M. Cochran S. D. D'Augelli A. R.. (2010). Suicide and suicide risk in lesbian, gay, bisexual, and transgender populations: Review and recommendations. J. Homosex. 58, 10–51. 10.1080/00918369.2011.534038 21213174 PMC3662085

[B27] Hatzenbuehler M. L. Birkett M. Van Wagenen A. Meyer I. H. (2014). Protective school climates and reduced risk for suicide ideation in sexual minority youths. Am. J. Public Health 104, 279–286. 10.2105/AJPH.2013.301508 24328634 PMC3935661

[B28] Hatzenbuehler M. L. Flores A. R. Gates G. J. (2017). Social attitudes regarding same-sex marriage and LGBT health disparities: results from a national probability sample. J. Soc. Issues 73, 508–528. 10.1111/josi.12229

[B29] Hatzenbuehler M. L. Nolen-Hoeksema S. Dovidio J. (2009). How does stigma “get under the skin”? The mediating role of emotion regulation. Psychol. Sci. 20, 1282–1289. 10.1111/j.1467-9280.2009.02441.x 19765237 PMC3687354

[B30] Hatzenbuehler M. L. Pachankis J. E. (2016). Stigma and minority stress as social determinants of health among lesbian, gay, bisexual, and transgender youth: research evidence and clinical implications. Pediatr. Clin. North Am. 63, 985–997. 10.1016/j.pcl.2016.07.003 27865340

[B31] Healthy People 2020 (2020). Healthy People 2020. Available online at: https://www.healthypeople.gov/ (accessed June 2021).

[B32] Heckathorn D. D. (2011). Comment: snowball versus respondent-driven sampling. Sociol. Methodol. 41, 355–366. 10.1111/j.1467-9531.2011.01244.x 22228916 PMC3250988

[B33] Hendricks M. L. Testa R. J. (2012). A conceptual framework for clinical work with transgender and gender nonconforming clients: an adaptation of the Minority Stress Model. Prof. Psychol. Res. Pract. 43, 460–467. 10.1037/a0029597

[B34] Katz-Wise S. L. Scherer E. A. Calzo J. P. Sarda V. Jackson B. Haines J.. (2015). Sexual minority stressors, internalizing symptoms, and unhealthy eating behaviors in sexual minority youth. Ann. Behav. Med. 49, 839–852. 10.1007/s12160-015-9718-z 26156678 PMC4636454

[B35] Kaufman T. M. Baams L. Veenstra R. (2020). Disparities in persistent victimization and associated internalizing symptoms for heterosexual versus sexual minority youth. J. Res. Adolesc. 30, 516–531. 10.1111/jora.12495 30927389 PMC7064905

[B36] Kosciw J. G. Greytak E. A. Bartkiewicz M. J. Boesen M. J. Palmer N. A. (2012). The 2011 National School Climate Survey: The Experiences of Lesbian, Gay, Bisexual and Transgender Youth in Our Nation's Schools. New York, NY: Gay, Lesbian and Straight Education Network.

[B37] Lang A. J. Wilkins K. Roy-Byrne P. P. Golinelli D. Chavira D. Sherbourne C.. (2012). Abbreviated PTSD checklist (PCL) as a guide to clinical response. Gen. Hosp. Psychiatry 34, 332–338. 10.1016/j.genhosppsych.2012.02.003 22460001 PMC3383936

[B38] Luk J. W. Gilman S. E. Haynie D. L. Simons-Morton B. G. (2018). Sexual orientation and depressive symptoms in adolescents. Pediatrics 141:e20173309. 10.1542/peds.2017-3309 29661939 PMC5931790

[B39] Marín O. (2016). Developmental timing and critical windows for the treatment of psychiatric disorders. Nat. Med. 22, 1229–1238. 10.1038/nm.4225 27783067

[B40] Marshal M. P. Dietz L. J. Friedman M. S. Stall R. Smith H. A. McGinley J.. (2011). Suicidality and depression disparities between sexual minority and heterosexual youth: a meta-analytic review. J. Adolesc. Health 49, 115–123. 10.1016/j.jadohealth.2011.02.005 21783042 PMC3649127

[B41] McGeough B. L. Sterzing P. R. (2018). A systematic review of family victimization experiences among sexual minority youth. J. Prim. Prev. 39, 491–528. 10.1007/s10935-018-0523-x 30206750 PMC6408293

[B42] Melchior L. A. Huba G. J. Brown V. B. Reback C. J. (1993). A short depression index for women. Educ. Psychol. Meas. 53, 1117–1125. 10.1177/0013164493053004024

[B43] Meyer I. H. (2003). Prejudice, social stress, and mental health in lesbian, gay, and bisexual populations: conceptual issues and research evidence. Psychol. Bull. 129, 674–697. 10.1037/0033-2909.129.5.674 12956539 PMC2072932

[B44] Meyer I. H. Bayer R. (2013). School-based gay-affirmative interventions: first amendment and ethical concerns. Am. J. Public Health 103, 1764–1771. 10.2105/AJPH.2013.301385 23948002 PMC3780740

[B45] Meyer I. H. Marken S. Russell S. T. Frost D. M. Wilson B. D. (2020). An innovative approach to the design of a national probability sample of sexual minority adults. LGBT Health 7, 101–108. 10.1089/lgbt.2019.0145 32130087 PMC7138603

[B46] Morrison M. A. Bishop C. J. Morrison T. G. (2018). A systematic review of the psychometric properties of composite LGBT prejudice and discrimination scales. J. Homosex. 66, 549–570. 10.1080/00918369.2017.1422935 29308989

[B47] Mossman S. A. Luft M. J. Schroeder H. K. Varney S. T. Fleck D. E. Barzman D. H.. (2017). The generalized anxiety disorder 7-item (GAD-7) scale in adolescents with generalized anxiety disorder: signal detection and validation. Ann. Clin. Psychiatry 29, 227–234. 29069107 PMC5765270

[B48] Muehlenkamp J. J. Hilt L. M. Ehlinger P. P. McMillan T. (2015). Nonsuicidal self-injury in sexual minority college students: a test of theoretical integration. Child Adolesc. Psychiatry Ment. Health 9, 1–8. 10.1186/s13034-015-0050-y 26157477 PMC4495628

[B49] National Academy of Medicine (2015). Annual Report 2015. Available online at: https://nam.edu/wp-content/uploads/2016/06/NAM-Annual-Report-2015.pdf (accessed June 2021).

[B50] Norris A. L. Orchowski L. M. (2020). Peer victimization of sexual minority and transgender youth: a cross-sectional study of high school students. Psychol. Violence 10, 201–211. 10.1037/vio0000260 35979532 PMC9380522

[B51] Onken L. Carroll K. Shoham V. Cuthbert B. Riddle M. (2014). Reenvisioning clinical science: unifying the discipline to improve the public health. Clin. Psychol. Sci. 2, 22–34. 10.1177/2167702613497932 25821658 PMC4374633

[B52] Pachankis J. E. Hatzenbuehler M. L. Rendina H. J. Safren S. A. Parsons J. T. (2015). LGB-affirmative cognitive-behavioral therapy for young adult gay and bisexual men: a randomized controlled trial of a transdiagnostic minority stress approach. J. Consult. Clin. Psychol. 83, 875–889. 10.1037/ccp0000037 26147563 PMC4573250

[B53] Padilla Y. C. Crisp C. Rew D. L. (2010). Parental acceptance and illegal drug use among gay, lesbian, and bisexual adolescents: results from a national survey. Soc. Work 55, 265–275. 10.1093/sw/55.3.265 20632661

[B54] Parker K. Morin R. Horowitz J. M. Lopez M. H. Rohal M. (2015). Multiracial in America: Proud, Diverse and Growing in Numbers. Washington, D.C.: Pew Research Center.

[B55] Petrocchi N. Pistella J. Salvati M. Carone N. Laghi F. Baiocco R. (2020). I embrace my LGB identity: self-reassurance, social safeness, and the distinctive relevance of authenticity to well-being in Italian lesbians, gay men, and bisexual people. Sex. Res. Soc. Policy 17, 75–86. 10.1007/s13178-018-0373-6

[B56] Poštuvan V. Podlogar T. Šedivy N. Z. De Leo D. (2019). Suicidal behaviour among sexual-minority youth: a review of the role of acceptance and support. Lancet Child Adolesc. Health 3, 190–198. 10.1016/S2352-4642(18)30400-0 30679139

[B57] Raifman J. Charlton B. M. Arrington-Sanders R. Chan P. A. Rusley J. Mayer K. H.. (2020). Sexual orientation and suicide attempt disparities among US adolescents: 2009–2017. Pediatrics 145:e20191658. 10.1542/peds.2019-1658 32041815 PMC7049939

[B58] Rice E. Barman-Adhikari A. (2014). Internet and social media use as a resource among homeless youth. J. Comput. Mediat. Commun. 19, 232–247. 10.1111/jcc4.12038 25328374 PMC4200536

[B59] Rosario M. Schrimshaw E. W. Hunter J. Gwadz M. (2002). Gay-related stress an emotional distress among gay, lesbian and bisexual youths: a longitudinal examination. J. Consult. Clin. Psychol. 70, 967–975. 10.1037/0022-006X.70.4.967 12182280

[B60] Ryan C. (2010). Engaging families to support lesbian, gay, bisexual, and transgender youth: the family acceptance project. Prevent. Res. 17, 11–13. 10.1037/e509042011-003

[B61] Ryan C. Huebner D. Diaz R. M. Sanchez J. (2009). Family rejection as a predictor of negative health outcomes in white and Latino lesbian, gay, and bisexual young adults. Pediatrics 123, 346–352. 10.1542/peds.2007-3524 19117902

[B62] Salvati M. Pistella J. Ioverno S. Laghi F. Baiocco R. (2018). Coming out to siblings and internalized sexual stigma: the moderating role of gender in a sample of Italian participants. J. GLBT Fam. Stud. 14, 405–424. 10.1080/1550428X.2017.1369916

[B63] Schrager S. M. Goldbach J. T. Mamey M. R. (2018). Development of the sexual minority adolescent stress inventory. Front. Psychol. 9, 1–16. 10.3389/fpsyg.2018.00319 29599737 PMC5862853

[B64] Schrager S. M. Mamey M. R. Rhoades H. Goldbach J. T. (2021). The adolescent stress experiences over time study (ASETS): a prospective longitudinal study of sexual minority adolescents in the United States. [Manuscript submitted for publication]. 10.1136/bmjopen-2021-054792 PMC8915334 35264352

[B65] Schwab-Reese L. M. Currie D. Mishra A. A. Peek-Asa C. (2021). A comparison of violence victimization and polyvictimization experiences among sexual minority and heterosexual adolescents and young adults. J. Interpers. Viol. 36, NP5874–NP5891. 10.1177/0886260518808853 30406715

[B66] Sterzing P. R. Ratliff G. A. Gartner R. E. McGeough B. L. Johnson K. C. (2017). Social ecological correlates of polyvictimization among a national sample of transgender, genderqueer, and cisgender sexual minority adolescents. Child Abuse Negl. 67, 1–12. 10.1016/j.chiabu.2017.02.017 28226283

[B67] Taliaferro L. A. Muehlenkamp J. J. (2017). Nonsuicidal self-injury and suicidality among sexual minority youth: risk factors and protective connectedness factors. Acad. Pediatr. 17, 715–722. 10.1016/j.acap.2016.11.002 28865597

[B68] Testa R. J. Habarth J. Peta J. Balsam K. Bockting W. (2015). Development of the gender minority stress and resilience measure. Psychol. Sex. Orient. Gend. Divers. 2, 65–77. 10.1037/sgd000008133224698

[B69] Toomey R. B. Ryan C. Diaz R. M. Card N. A. Russell S. T. (2013). Gender-nonconforming lesbian, gay, bisexual, and transgender youth: School victimization and young adult psychosocial adjustment. Dev. Psychol. 46, 1580–1589. 10.1037/a0020705 20822214

[B70] Tyler K. A. Ray C. M. (2019). Risk and protective factors for substance use among youth experiencing homelessness. Child. Youth Serv. Rev. 107:104548. 10.1016/j.childyouth.2019.104548 31827311 PMC6905194

[B71] U. S. Census Bureau (2012). Growth in Urban Population Outpaces Rest of Nation, Census Bureau Reports. Available online at: https://www.census.gov/newsroom/releases/archives/~2010_census/cb12-50.html (accessed June 2021).

[B72] Watkins C. E. Campbell V. L. Nieberding R. Hallmark R. (1995). Contemporary practice of psychological assessment by clinical psychologists. Prof. Psychol. Res. Pract. 26, 54–60. 10.1037/0735-7028.26.1.54

[B73] Weeks S. N. Renshaw T. L. Galliher R. V. Tehee M. (2020). The moderating role of psychological inflexibility in the relationship between minority stress, substance misuse, and suicidality in LGB+ adolescents. J. Context. Behav. Sci. 18, 276–286. 10.1016/j.jcbs.2020.10.007

[B74] Willging C. E. Salvador M. Kano M. (2006). Unequal treatment: mental health care for sexual and gender minority groups in a rural state. Psychiatr. Serv. 57, 867–870. 10.1176/ps.2006.57.6.867 16754766

[B75] Wolchik S. Sandler I. N. (2013). Handbook of Children's Coping: Linking Theory and Intervention. Washington, D.C.: Springer Science & Business Media.

