# Supplementary material for: Confirming the Reliability and Validity of the Sexual Minority Adolescent Stress Inventory in a National Sample of Sexual Minority Adolescents
Source: Front Psychol. 2021 Aug 31;12:720199. doi: 10.3389/fpsyg.2021.720199 (PMC8438190; doi:10.3389/fpsyg.2021.720199)
Supplement: Supplementary file 1 [file Table_1.docx]

|  | | | | | | | | |
| --- | --- | --- | --- | --- | --- | --- | --- | --- |
|  | Depression | Anxiety | PTSD | Ideation | Plan | Attempt | Self-harm |  |
| TS: Total score SMASI | .326** | .346** | .420** | .370** | .366** | .311**** | .298** |  |
| 1: Identity management | .140** | .149** | .152** | .151** | .165** | .068* | .190** |  |
| 2: Negative expectancies | .212** | .240** | .242** | .245** | .249** | .166** | .216** |  |
| 3: Negative disclosure experiences | .190** | .191** | .231** | .214** | .225** | .233** | .175** |  |
| 4: Family rejection | .259** | .265** | .330** | .281** | .276** | .243** | .210** |  |
| 5: Internalized homonegativity | .095** | .099** | .128** | .128** | .123** | .056 | .097** |  |
| 6: Homonegative communication | .254** | .273** | .312** | .299** | .269** | .183** | .235** |  |
| 7: Homonegative climate | .216** | .272** | .307** | .266** | .245** | .221** | .230** |  |
| 8: Social marginalization | .224** | .245** | .310** | .270** | .271** | .268** | .235** |  |
| 9: Intersectionality | .183** | .169** | .246** | .188** | .196** | .174** | .116** |  |
| 10: Religion | .230** | .230** | .308** | .232** | .247** | .256** | .185** |  |
| 11: Work | .181** | .176** | .234** | .213** | .216** | .216** | .150** |  |

|  | | |  | |  |  |  |  |  |  |
| --- | --- | --- | --- | --- | --- | --- | --- | --- | --- | --- |
|  | Alc (L) | Mar(L) | | Presc (L) | | Tob (L) | Alc (D) | Mar (D) | Presc (D) | Tob (D) |
| TS: Total score SMASI | .182** | .164** | | .241** | | .191 | .207** | .141** | .243** | .193** |
| 1: Identity management | .072* | .062* | | .118** | | .061 | .098* | .039 | .089* | .085* |
| 2: Negative expectancies | .039 | -.014 | | .059 | | .064 | .076* | .001 | .136* | .085* |
| 3: Negative disclosure experiences | .211** | .183** | | .217** | | .193 | .210** | .154** | .203** | .170** |
| 4: Family rejection | .099** | .075* | | .156** | | .094 | .114** | .079* | .147** | .068* |
| 5: Internalized homonegativity | .080* | .077* | | .090 | | .039 | .090* | .052 | .089* | .052 |
| 6: Homonegative communication | .195** | .163** | | .186 | | .186 | .186** | .147** | .174** | .187** |
| 7: Homonegative climate | .139** | .123** | | .168 | | .228 | .172** | .110** | .225** | .240** |
| 8: Social marginalization | .146** | .166** | | .188 | | .173 | .164** | .139** | .158** | .185** |
| 9: Intersectionality | .054 | .105** | | .116 | | .066 | .102** | .100* | .143* | .060 |
| 10: Religion | .067* | .076* | | .181 | | .104 | .061* | .042 | .163** | .110* |
| 11: Work | .092* | .092* | | .166 | | .136 | .107* | .091* | .206** | .114* |
